# Reducing the critical particle diameter in (highly) asymmetric sieve-based lateral displacement devices

**DOI:** 10.1038/s41598-017-14391-z

**Published:** 2017-10-26

**Authors:** J. P. Dijkshoorn, M. A. I. Schutyser, M. Sebris, R. M. Boom, R. M. Wagterveld

**Affiliations:** 1 Laboratory of Food Process Engineering, Wageningen University, Bornse Weilanden 9, 6708WG Wageningen, The Netherlands; 2 Wetsus, European Centre of Excellence for Sustainable Water Technology, Oostergoweg 9, 8911MA Leeuwarden, The Netherlands

## Abstract

Deterministic lateral displacement technology was originally developed in the realm of microfluidics, but has potential for larger scale separation as well. In our previous studies, we proposed a sieve-based lateral displacement device inspired on the principle of deterministic lateral displacement. The advantages of this new device is that it gives a lower pressure drop, lower risk of particle accumulation, higher throughput and is simpler to manufacture. However, until now this device has only been investigated for its separation of large particles of around 785 µm diameter. To separate smaller particles, we investigate several design parameters for their influence on the critical particle diameter. In a dimensionless evaluation, device designs with different geometry and dimensions were compared. It was found that sieve-based lateral displacement devices are able to displace particles due to the crucial role of the flow profile, despite of their unusual and asymmetric design. These results demonstrate the possibility to actively steer the velocity profile in order to reduce the critical diameter in deterministic lateral displacement devices, which makes this separation principle more accessible for large-scale, high throughput applications.

## Introduction

**Figure 1:**
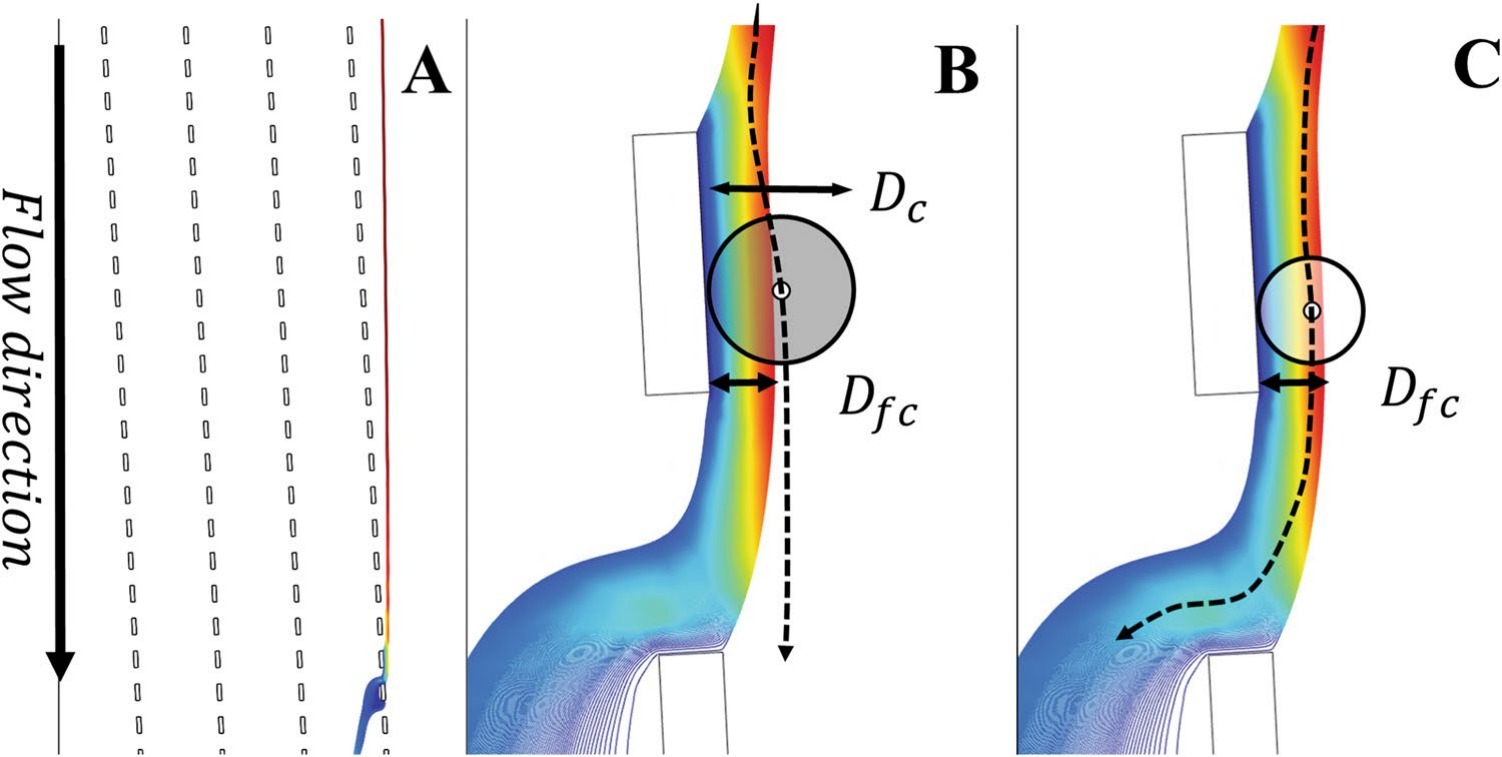
Visual representation of a fluid flow lane through a gap between white obstacles, derived from a 2D simulation. The blue colour represents low velocity; red colour a high velocity. The layout of a device having 4 obstacle columns is shown in (**A**). In (**B**) the gray circle represents a particle larger than the critical diameter being displaced and in (**C**) the white circle represents a small particle that stays in the flow lane and follows the flow direction.

Deterministic lateral displacement technology is originally a microfluidic suspension separation technique that holds potential for large scale separation of suspensions: it features low pressure drop and low risk of particle accumulation while the design and operation is simple^1–7^. Deterministic lateral displacement devices exploit arrays of obstacles in which each row is slightly displaced relative to the previous row. The fluid that flows between two obstacles in subsequent rows, is called a flow lane (Fig. 1). When the radius of a particle is larger than the width of its flow lane (D_fc_), the particle will be displaced laterally. Due to its hydrodynamic interaction with the obstacle it moves into the next flow lane (Fig. 1B). Particles having a diameter smaller than the critical diameter (D_c_) are not displaced. Instead they follow the direction of the fluid flow (Fig. 1C) and pass through the array of obstacles along with the fluid^8^. Eventually this leads to separation or fractionation of particles that are different in size.

**Figure 2:**
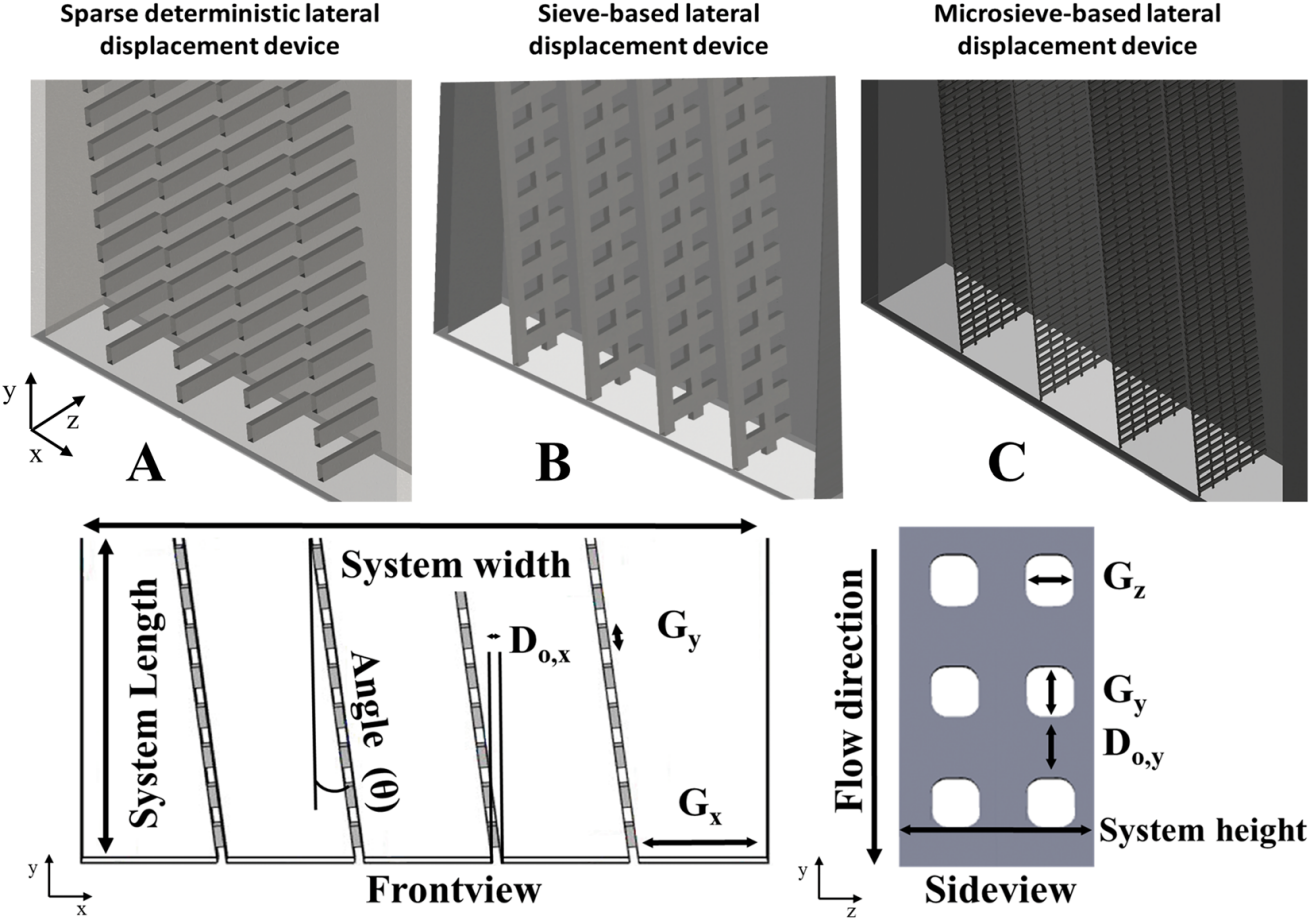
3D representation of (**A**) a sparse deterministic lateral displacement device^10^, (**B**) a sieve-based lateral displacement device^11^ and (**C**) a sieve-based lateral displacement device that employs micro sieves for separation of smaller particles. In (**D**) an overview of important geometric parameters in these devices. The exact parameter values for each system are shown in Table 2.

The highest reported throughput for microfluidic separation in a single deterministic lateral displacement device is approximately 2 mL/min^9^. For analytical purposes this is large enough, but it is far too small for large-scale industrial separation applications^5^. To enable larger-scale separation, sparse obstacle array designs with lower numbers of obstacles (up to 90% less) have been proposed^10^. The sparse designs are characterised by a lower pressure drop, reduced risk of fouling and easier scale-up^10^. Another advantage of the sparse design is simpler construction of obstacles by applying sieves instead of manufacturing individual pillars (Fig. 2). In previous research we reported on particle displacement of relatively large particles with a D50 of 785 µm with sparse and sieve-based lateral displacement devices^10,11^.

Here, we aim to decrease the size of particles that can be separated while maximizing the throughput using the sieve-based lateral displacement devices. The concept is to employ micro-sieves in order to separate small particles that are closer to industrially relevant suspensions. Eventually the ambition is to separate cells, algae or starch granules with a size between 5 and 20 µm, although in this study the smallest particles have a diameter between 70 and 140 µm. To effectively separate small particles the critical particle diameter and thus flow lane needs to be small. The size of the flow lanes does not only depend on the size of the pores in the sieves but depends on more (geometrical) parameters. For this reason the deterministic lateral displacement theory (Equation 1) was analysed^3,12^.

1 $${D}_{c}=2\alpha \,\sin \,\theta \frac{({G}_{y}+{D}_{o,y})}{{G}_{x}+{D}_{o,x}}{G}_{x}$$

where D_c_ is the critical particle diameter, α is a dimensionless correction factor for a non-uniform flow profile, θ is the angle in which the sieves are placed, G_y_ is the gap in downstream direction, G_x_ is the lateral gap and D_o_ the obstacle size in x or y direction.

Note that the geometric parameters and operational conditions of the sieve-based systems used in this study vary (open design and unequal outflow). This results in varying G_x_ and α along the length (y) of the device and a critical diameter that depends on location. Moreover, because α might not be completely independent of other parameters, it is not possible to estimate the critical diameter with theory described above and thus should be derived from experiments or numerical simulations. Nonetheless, Equation 1 introduces crucial parameters to scale down the critical diameter in full deterministic lateral displacement arrays, namely *α*,θ,*G*_*y*_,*G*_*x*_ and *D*_*O*_. Accordingly we study these parameters for their influence on the critical particle diameter in sieve-based lateral displacement devices (except for D_o_ because of practical reasons). The influence of these parameters on suspension separation provides guidelines towards a system design in which the dimensions are specifically adjusted for high throughput separation purposes.

## Materials and Methods

### Materials

Particle suspensions were prepared with demineralized water, 0.1 w/v% Tween-80 (Merck, Germany) and 0.04 v/v% polyethylene particles (Cospheric, USA). These particles have a density of 0.98–1.00 g/ml. The particle size distribution was measured with a Mastersizer 2000 (Malvern, UK), shown in Table 1.

**Table 1:** Particle size distribution in µm.

Particles	D10	D50	D90
Small	54	73	98
Medium	76	102	137
Large	104	140	189

### Devices

**Figure 3:**
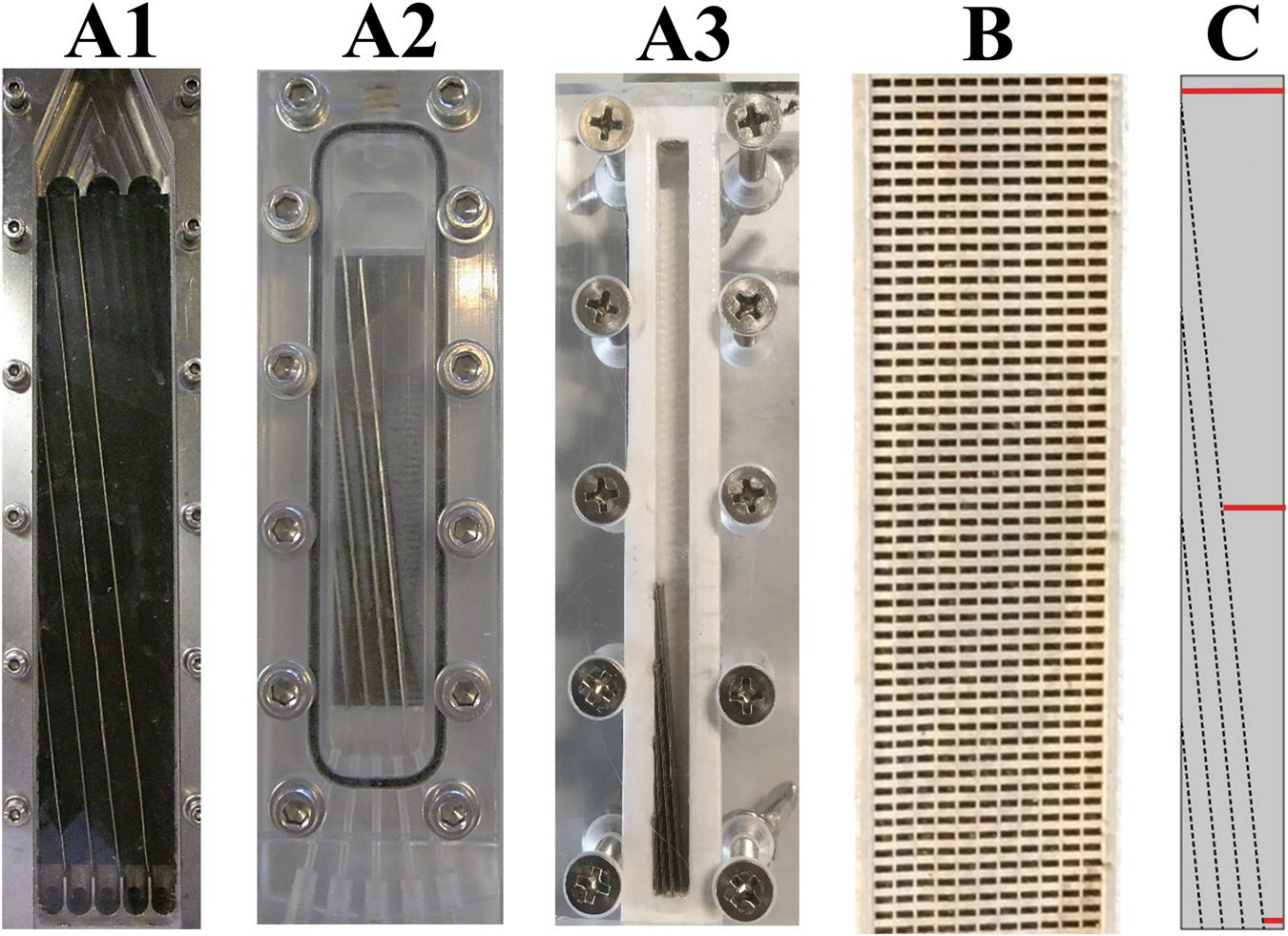
(**A**) System 1 is the largest flow cell^21,22^, System 2 is an intermediate sized flow cell previously used by Lubbersen *et al*.^13^ and system 3 the smallest flow cell. (**B**) shows the sieve (nickel) used to serve as obstacles inside the devices (pores are black). (**C**) shows the geometry of system 3 only, system 1 and 2 are shorter and therefore the sieves do not start on the left hand sidewall. The red lines at the top middle and bottom are the locations where the velocity profiles are taken (Fig. 7). The geometrical parameters are given in Table 2.

The influence of θ, G_y_ and G_x_ was investigated using three different sized flow devices (Fig. 3A), with system 1 being the largest and system 3 the smallest. The design and parameters of all used devices are given in Figs 2, 3 and Table 2. Systems 1 and 2 were previously used by Lubbersen and co-workers and also employed for this study^13^. Both systems 1 and 2 (Fig. 3A) have a base plate with grooves of 2mm depth (fine milled polyoxymethylene) in which the sieves are positioned. In these two systems the two most right sieves do not touch the left wall.

**Table 2:** Geometrical parameters of the systems. The number of pores in depth of the system (sieve) is indicated behind the depth of an individual gap in brackets.

	Angle [θ]	G_y_ [mm]	G_x_ [mm]	G_z_ [mm]	D_o,x_ [mm]	D_o,y_ [mm]	Width [mm]	Height [mm]	Length [mm]	Porosity [%]
System 1	5.9	0.2	8.9	0.5 (9x)	0.05	0.2	44.8	5	216	~45
System 2	5.9	0.2	2.2	0.5 (9x)	0.05	0.2	11.2	5	5.4	~45
System 3	2.9–5.9	0.2	1	0.5 (11x)	0.05	0.2	5	7	10	~45

The lateral gap size is variable and therefore the smallest lateral gap is indicated for comparison.

System 3 was constructed from polylactic acid (PLA) with a 3D printer (Ultimaker 2+, The Netherlands). The five outlets are constructed with injection needles with an outer diameter of 0.7 mm for outlet 1 to 4 and 1.3 mm for outlet 5. In order to investigate θ, four versions of system 3 were constructed with varying sieve configurations (Table 2). This was done because the sieves in this system are permanently fixed. For all systems, the suspension is introduced (Masterflex L/S, Cole-Palmer, Chicago, IL) from the top and collected at the five outlets at the bottom.

Figure 3B shows one of the sieves used, where the pores (black) are 200 by 500 µm and the support structures between the pores (nickel) are 200 µm in vertical direction and 50 µm in horizontal direction. The sieves have post aspect ratios of 25 (system 1 and 2) and 35 (system 3), which is about 4 to 17 times larger than reported previously^4,13^.

### Experimental procedures

Experiments with varying geometrical parameters were performed with the three systems described in Table 2. For the experiments, suspensions with three different size particles sizes (Table 1) were used to determine the critical diameter. All experiments were conducted in triplicate (n = 3) with an average inlet flow velocity of 0.12 m/s. In addition, these systems are operated with adjusted outflow conditions to ensure the optimal pressure distribution (Table 3)^11^. These conditions were selected based on experimental observations of particle trajectories. The inlet concentration was calculated using the weighted average of the outlet concentrations and volumetric outflow rates^11^. To enable comparison between the different experiments, the outlet concentrations are normalized with the measured inlet concentration (0.04 v/v%), because the inlet concentration may vary slightly per experiment. The experimental concentrations are presented as mean ± standard deviation (SD) and were analysed using one-way analysis of variance (ANOVA) and Welch’s t-test.

**Table 3:** Experimental outlet conditions in percentages used while operating the different systems.

	Outlet 1	Outlet 2	Outlet 3	Outlet 4	Outlet 5
System 1	16%	16%	16%	16%	36%
System 2	16%	16%	16%	16%	36%
System 3	18%	18%	18%	18%	28%

System 3 was investigated for four different angles (5.9°, 4.9°, 3.9° and 2.9°) with the same outflow conditions.

### Numerical simulations

COMSOL Multiphysics 5.2a was used to create 2D models of the 3 systems described in Fig. 3. Figure 3C shows an example of one of the devices and the locations were the velocity profile are taken. The fluid was considered to be incompressible, stationary in the laminar regime and had the physical properties of water at 293.15 K. The laminar inlet flow was parabolic with an average velocity of 0.12 m/s. Outlets 1 to 4 were fixed at specific outflow conditions and the outflow from outlet 5 was based on pressure. A no-slip boundary condition was applied. A mesh dependency study was performed and the results were independent of mesh size.

## Results and Discussion

### Separation with varying sieve angle (θ)

**Figure 4:**
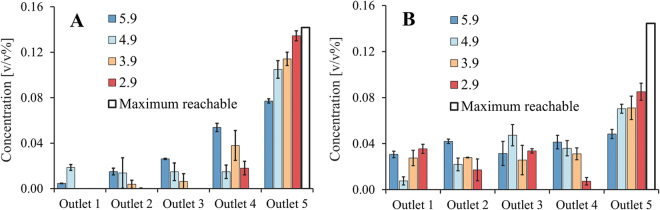
Comparison of experimental concentration (mean ± 1 SD) with variable sieve angle (θ) in system 3 and the maximal concentration that can be reached in theory (white). For all experiments the average inlet velocity was 0.12 m/s and the inlet suspension contained 0.04 v/v% particles. In (**A**) the results of large particles with a D50 of 140 µm are shown and in (**B**) the results of small particles with D50 of 102 µm. The concentrations in outlet 5 are significantly different for both particle sizes (one-way ANOVA: p < 0.005), therefore there is an effect of the angle on concentrations.

First we describe the influence of the sieve angle (θ) on separation. For this investigation four systems were prepared, in each system the sieves are placed at a different angle. The white bar indicates the maximum reachable concentration, when all particles are displaced and end up in outlet 5. The results presented in Fig. 4 show two suspensions with large (A) and medium (B) sized particles (Table 1) and demonstrate an increasing particle concentration in outlet 5 with decreasing angle. The mean concentrations measured in outlet 5 are significantly influenced by changing the sieve angle (one-way ANOVA: p < 0.005), which holds for both particle sizes. These results agree with observations done for conventional full deterministic lateral displacement systems^12,14^.

The results in Fig. 4A show that the system with sieves placed at an angle of 2.9°, recovers 95% of all large particles in the targeted outlet. A very low angle may cause practical limitations, since systems with considerable displacement will have to be relatively long^12^. Longer systems usually also exhibit a larger pressure drop, but in case of open sparse systems this is of less concern.

Additional experiments were carried out with medium sized particles (Fig. 4B). The smaller particles show a similar trend compared to the larger particles in Fig. 4A. The separation improves with decreasing angle, but the concentrations in outlet 5 are lower compared to the experiment with larger size range.

A decreasing angle leads to a reduction in the critical diameter and thus an increase in concentration of outlet 5 could be expected: the system with an angle of 2.9 concentrates 57% of all particles in outlet 5.

When the results presented in Fig. 4 are combined with the particle size distributions (Table 1) the critical diameter can be estimated to be between 100–140 µm for angles between 2.9° and 5.9°. This can be derived from the observation that the system with an angle of 5.9° separates around half of the particles with a D50 of 140 µm, but hardly any particles with a D90 of 137 µm. When the angle is 2.9° nearly all particles with a D10 of 104 µm are separated and about 57% of the particles with a D50 of 102 µm. These results illustrate the influence of the sieve angle on the critical diameter in sieve-based lateral displacement systems.

### Gap in downstream direction (G_y_) and system overview

**Figure 5:**
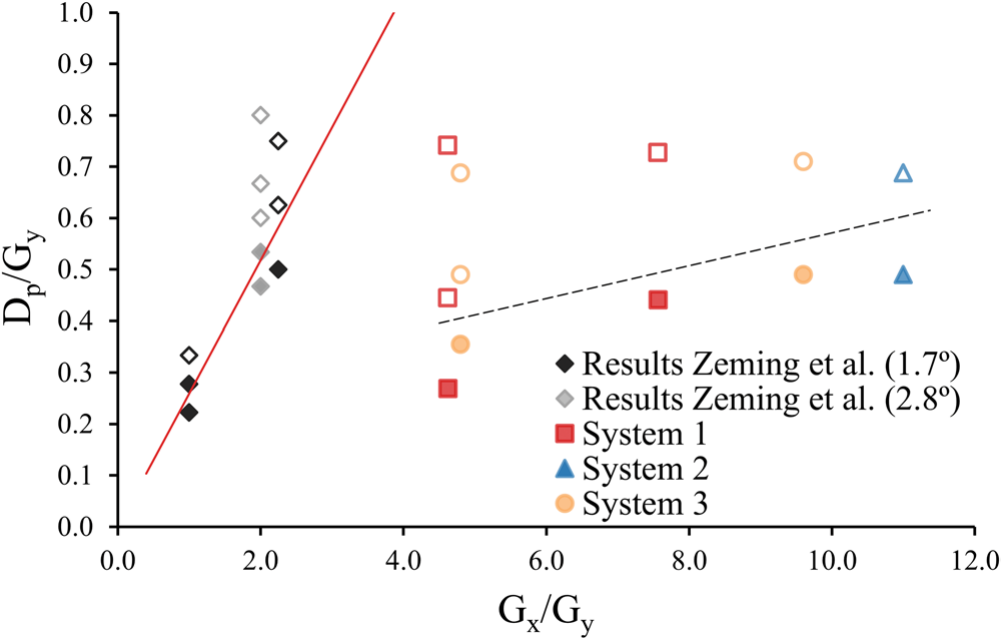
System asymmetry versus fraction of filtration limit; dimensionless comparison of Gy relative to Gx and Dp. The experimental results are obtained with different (sized) sparse, sieve-based systems and deterministic lateral displacement devices3, 10, 11. The open marks indicate particle displacement, this implies that these measurements had a significantly higher concentration in outlet 5 compared to the inlet concentration (one sample Welch’s t-test: p < 0.05). The filled marks indicate that particles were not displaced, which means no significant difference was observed between the inlet concentration and the concentration in outlet 5 (one sample Welch’s t-test: p < 0.05). The black and gray diamonds illustrate the results of a full obstacle array with an θ of 1.7° and 2.8° respectively 3. The red line indicates the estimated critical diameter for the full deterministic lateral displacement devices with a θ of 1.7°, using the empirical model of Davis *et al*.^23^. The black dotted line guides the eye and shows the distinction between separation and no separation for the sparse systems with different proportions, an angle of 5.9° and an average inlet velocity of 0.12 m/s.

The G_y_ size is known to influence successful separation as well^3^. Because different system designs (Table 4) and particle sizes are compared here, the G_y_ is made dimensionless by relating it to G_x_ and the mean particle diameter of the suspension (D_p_). In this work, the D_p_ is related to the G_y_ instead of the usually used G_x_^12^. The reason is that for the discussed systems G_y_ is the smallest gap and determines whether particles are displaced or filtered; while for most deterministic lateral displacement systems G_x_ is limiting. For the overview in Fig. 5, several systems of Zeming *et al*.^3^ as well as the sparse and sieve-based systems are analysed. The geometrical parameters of the sparse and sieve-based systems are described in Table 4.

**Table 4:** Geometrical parameters of the systems shown in Fig. 5.

	Angle [θ]	G_y_ [mm]	G_x_ [mm]	G_z_ [mm]	D_o,y_ [mm]	D_o,x_ [mm]	Width [mm]	Height [mm]	Length [mm]	Porosity [%]
System 1	5.9	1.8	8.3	1.5 (2x)	1.6	0.8	44.8	5	216	~50
	5.9	1.1	8.4	2.5	0.68	0.68	44.8	2.5	216	~62
	5.9	0.2	8.9	0.5 (9x)	0.2	0.05	44.8	5	216	~45
System 2	5.9	0.2	2.2	0.5 (9x)	0.2	0.05	11.2	5	5.4	~45
System 3	5.9	0.2	1	0.5 (11x)	0.2	0.05	5	7	10	~45
	5.9	0.1	1	0.5 (11x)	0.1	0.05	5	7	10	~45

The number of pores in depth of the system (sieve) is indicated behind the depth of an individual gap in brackets.

The results in Fig. 5 give an impression of the differences between the devices with regards to particle displacement as a function of the systems geometry. The criterion used for the data points to distinguish whether particles were displaced or not is based on the mean particle diameter and the particle concentration in the target outlet (outlet 5), which must be significantly higher than the inlet concentration (one sample Welch’s t-test: p < 0.05).

**Figure 6:**
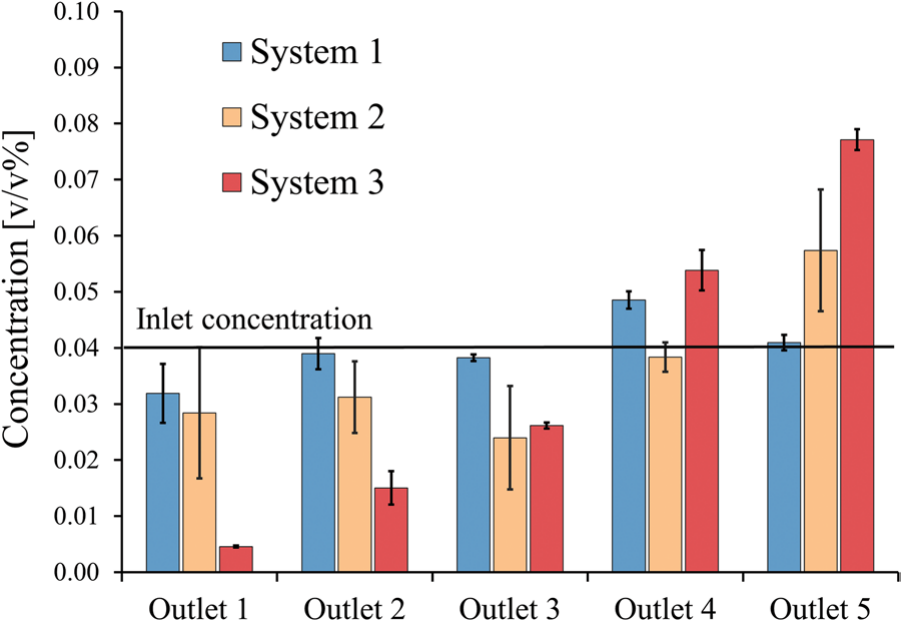
Particle concentrations (mean ± 1 SD) are shown per outlet for the three systems with varying in size and Gx. System 3 has different design compared to system 1 and system 2 (Fig. 3). The sieves (Fig. 3B) are identical and positioned at an angle of 5.9° in all three systems. The average inlet velocity was 0.12 m/s and the suspension contained 0.04 v/v% particles with a D50 of 140 µm. One-way ANOVA was performed on the concentrations of outlet 5 and the mean concentrations were found to significantly differ (p < 0.005).

The ratio of G_x_/G_y_ describes the asymmetry of the designs. Asymmetry (G_x_/G_y_>1) is desired since it reduces the pressure drop, the risk of particle accumulation and allows for effective upscaling^3,11^. However, in Figs 5 and 6 it can be observed that for fixed operational conditions the degree of asymmetry (G_x_/G_y_) is limiting and should not exceed a critical value specific for these conditions. If this critical value is crossed, D_p_ equals G_y_ which means that particles can no longer move through the gap and will cause obstruction and internal fouling. In the best situation, the D_p_ is much smaller than the G_y_ while it is still being displaced.

One should bear in mind that the systems of Zeming *et al*. are very different from the asymmetric systems described here. Not only the open design but also the obstacle shape/size, the inlet velocity and outflow conditions are different and thus can only be compared qualitatively. Regardless, all these systems are able to displace particles and it gives a perspective of the possibilities of using asymmetric systems.

The differences between the designs of the full deterministic lateral displacement devices and sparse lateral displacement devices are illustrated in Fig. 5. Full obstacle arrays are generally symmetric or moderately asymmetric and have a G_y_ that is 1 to 2.5 times as smaller as the G_x_. Asymmetric systems where G_y_ is bigger than G_x_ (ratio smaller than 1) have a larger pressure drop and a higher risk of clogging^3^, therefore they are not suitable for large scale and not taken into account. These full obstacle arrays are able to displace particles 2 to 3 times smaller than the downstream gap^3^.

Sparse or sieve-based lateral displacement systems are very asymmetric and have a G_y_ that is 4 to 11 times as smaller as the G_x_ but are still able to displace particles ~2 times smaller than the G_y_. That these systems, despite the extreme geometry are able to displace particles is possible because of the adjusted outflow conditions as was described earlier by Dijkshoorn *et al*. (2017). It is hypothesized that by changing the outflow conditions the uniformity of the flow profile is affected (α in Equation 1) such that it becomes possible to displace particles in systems with extreme geometry. However, the consequence of adjusting the outflow conditions, is a lower maximum attainable concentration in the target outlet^11^.

### Influencing α by varying the lateral gap (G_x_)

For a better understanding of α (the flow profile correction factor) and how it influences particle separation, the deterministic lateral displacement theory was applied to sparse and sieve-based lateral displacement systems. From this theory (Equation 1) it can be derived that when the geometry is very asymmetric (D_o,x_ ≪ G_x_); the influence of G_x_ (and D_o,x_) on the critical diameter becomes very small and can be neglected. This leads to Equation 2:

2 $${D}_{c}=2\alpha \,\sin \,\theta ({G}_{y}+{D}_{o,y})$$

On the basis of Equation 2, it is possible to change G_x_ without affecting D_c_. However, the results in Fig. 5 indicate that there is an effect of changing G_x_ relative to G_y_ (e.g. a higher ratio of D_p_/G_y_ can be observed with increasing G_x_/G_y_). It is hypothesized that G_x_ affects the flow or velocity profile (α), which is known to influence the critical particle diameter and thus separation^12,15,16^.

The velocity profile was investigated by systematically varying G_x_ in three different sized system designs (described in Fig. 3 and Table 2). These were operated with same sieve configuration and pore sizes (Fig. 3B), equal particle suspension (D_50_ of 140 µm) and equal average inlet velocity. The experimental results are shown in Fig. 6.

A significant increase in concentration can be observed for outlet 5 with decreasing system width or G_x_ (one-way ANOVA: p < 0.005). For the largest system 1, a somewhat higher concentration in outlet 4 was observed compared to the concentration in outlet 5. Overall however, limited particle displacement was observed for this system (supplementary movie). System 2 that has a width ~4 times smaller than system 1 reached a concentration of 0.057 v/v% in outlet 5. System 3, which is about two times narrower than system 2 obtained a mean concentration of 0.077 v/v%. A remark here is that the sieves in system 3 continue until the left border, unlike system 1 and 2 where the sieves stop short (Fig. 3A). As a result more particles are available for outlet 5 in system 3, which makes it difficult to compare system 3 with system 1 and 2. Moreover, system 3 was operated with different outflow conditions which were selected after experimental observations, because these conditions were found to improve pressure distribution for system 3.

Despite these differences, system 1 does not show separation but both systems 2 and 3 do show elevated concentrations in outlet 5. System 3 shows better depletion of outlet 1 and the highest concentration in outlet 5. This gives the impression that separation indeed is influenced by differences in G_x_ and possibly affects the velocity profile.

For confirmation, two-dimensional numerical flow simulations of the three systems (Fig. 3A) were created to illustrate the differences in the velocity profiles in the normalized lateral gap, and to visualize the changes in velocity profile along the length of the system at three locations (top, middle and bottom, Fig. 3C). Two-dimensional numerical simulations were chosen to reduce the computational requirements.

**Figure 7:**
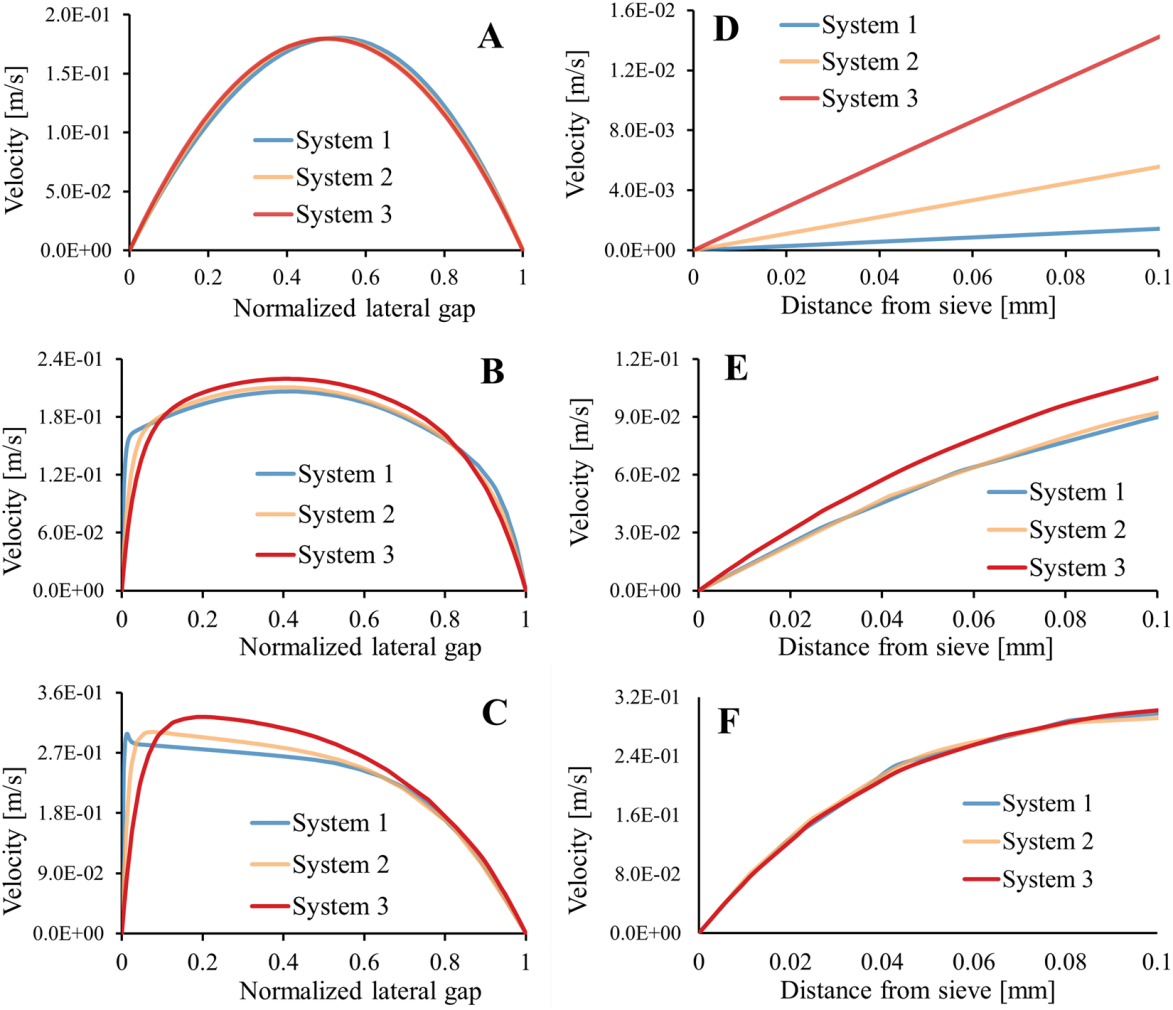
2D numerical simulations show the horizontal velocity profile at the top (AD), in the middle (BE) and at the bottom (CF, near outlet 5) of the three systems. ABC show the velocity profile over the normalized lateral gap and DEF show the first 100 μm starting from the sieve towards the channel centre. The results presented in were obtained by assuming similar outlet conditions for all systems with 36% in outlet 5 and 16% in the other outlets.

The velocity profiles (Fig. 7A–C) were set to start with the same parabolic shape. Progressing along the length of the system, the velocity profiles become more non-uniform under influence of a receding G_x_. Clear differences can be observed in the development of the velocity profile for the different systems.

**Figure 8:**
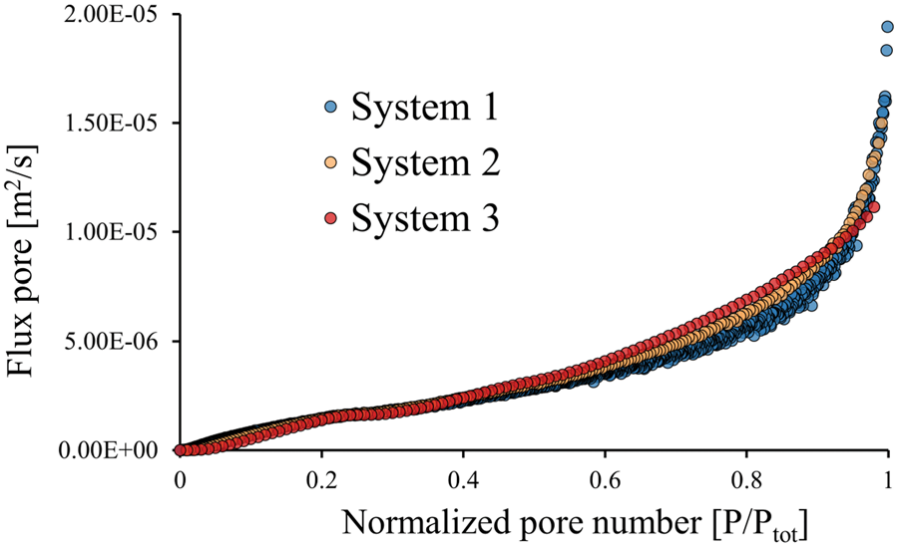
Numerically calculated flux through all pores of the three systems are shown, each marker indicates the flux in a single pore. For these results the same outflow conditions were used in all systems (outlet 1–4 at 16% and outlet 5 36%). The blue markers (system 1) have a larger spread because the mesh relative to the pores was larger compared to the other systems. However, improving the mesh did not affect the overall trend of the flux through the pores.

Figure 7D–F show the velocity profiles for the first 100 µm from the sieve towards the channel centre. Only the first 100 µm is shown because the gap in the downstream direction is 200 µm and a flow lane larger than 100 µm would result in a critical diameter larger than the gap. The velocity profile close to the sieve for the three systems (Fig. 7D–F) differ most at the top of the systems (D), where the smallest system shows the highest velocity gradient. In the middle of the systems (E) the differences in the velocity gradients have become smaller where system 2 and 3 became practically equal to each other. Near the bottom of the system (F) the velocity profiles close to the sieve are equal for all systems. A sharper velocity gradient will result in a somewhat smaller critical diameter assuming that the flow lanes carry equal flux, explaining the better performance of smaller systems^12,15,16^. Surprisingly, the velocity profile at the bottom and near the sieves become equal for all systems, which means that the smaller systems lose their advantage. A possible explanation is that the flow lanes do not carry equal flux along the length of the sieve and that flux of the flow lanes is larger for larger systems. Therefore, the same 2D models were used to verify the flux through the gaps over the entire length of the three systems (Fig. 8).

The calculations shown in Fig. 8 nicely illustrates that the flux through the gaps increases in the flow direction for all systems. This is different from conventional deterministic lateral displacement devices, where it is assumed that the flow lanes carry equal flux^8,12^. This assumption, however, is not valid for this system and not necessarily valid for systems with anisotropic permeability^17–20^. From the flux through the gaps (Fig. 8) and the partial area of the velocity profiles at these specific locations (Fig. 7D–F) it is possible to calculate the width of the flow lanes. These estimated flow lanes are shown in Table 5 and are in good agreement with the radius of the experimentally used particles (Table 1).

**Table 5:** Width of the flow lanes calculated by integrating the areal velocity profiles given in Fig. 7D–F and equalize it with the flux through the gaps (Fig. 8).

	Top	Middle	Bottom
System 1	6.7 µm	75.0 µm	96.2 µm
System 2	8.7 µm	72.3 µm	81.0 µm
System 3	3.9 µm	65.9 µm	66.3 µm

The flow lanes in the upper part of all systems are 22–50 times smaller than the gaps and become larger in the downstream direction. In system 1 and 2 the flow lanes become larger and their sizes ultimately increase up to 96 µm and 81 µm respectively. This increase is caused by the strong increase of the flux through the pores near the end of these systems (Fig. 8), which can be a result of the outflow conditions. The flow lanes in system 3 seem to stabilize around 66 µm in the middle and the bottom part which indicates a well-adjusted outflow condition. It is noted that the flow distribution results derived from the simulations do not correspond to the experimental results, where in systems 1 and 2 similar outlet flow conditions lead to optimal for flow distribution and surprisingly in system 3 even different outlet flow conditions were found optimal for flow distribution. These remarkable differences may be explained by the different flow distribution in a 2D simulation compared to flow in a 3D device in practice.

Extensive analyses shows that the velocity profile in these systems change substantially along the length of the axis, both in magnitude and in shape (Fig. 7). These changes in turn, affect the size of the flow lanes and determine the change in critical diameter along the length of the system. This uncertainty makes it impossible to obtain a single description for the critical particle diameter. However, the critical particle diameter might be estimated by considering the separation data in a dimensionless diagram for this specific angle (Fig. 5). Or alternatively, the minimum required G_x_ may be estimated for a chosen D_p_ and G_y_ for the specific inlet velocity. For example, to separate particles of 10 µm with a G_y_ of 20 µm, an angle of 5.9° and an average inlet velocity of 0.12 m/s, the smallest G_x_ should not be more than ~8 times the G_y_, i.e. 160 µm. These dimensions were cross-checked using COMSOL and found to be in good agreement.

## Conclusions

Design parameters of sparse and sieve-based lateral displacement systems were investigated for their influence on the critical particle diameter; with perspective of applying these systems for practically relevant large-scale application. The design and operation conditions of these systems are different from those of the full deterministic lateral displacement devices. For a better understanding of the interrelation between critical device parameters on the particles that can be separated, the parameters were varied systematically. The angle in which the sieves are placed influences the critical diameter in sparse obstacle arrays, which is in agreement with previous findings based on the existing deterministic lateral displacement technology. Highly asymmetric lateral displacement systems with a much smaller downstream gap (G_y_) than the lateral gap (G_x_) proved to be able to displace particles ~2 times smaller than the downstream gap (G_y_). Based on theory it might be expected that in highly asymmetric systems the lateral gap (G_x_) has little influence on the critical diameter; however, it was found that G_x_ has indirect influence on the critical diameter by influencing the hydrodynamics in these systems. Moreover, asymmetric lateral displacement systems are only able to displace particles, because the velocity profile becomes increasingly non-uniform (α) and stabilizes with increasing flux through the pores. These results show the possibilities to use the deterministic lateral displacement separation principle by actively governing the hydrodynamics instead of being restricted by the geometry. Because of the geometric and hydrodynamic differences compared to the full deterministic lateral displacement devices, it is not possible to estimate an overall critical particle diameter. However, it is possible to make a dimensionless comparison of different systems to approximate the required dimensions (e.g. lateral gap (G_x_) and downstream gap (G_y_)) for the specific operation conditions and particle diameter.

### Data availability statement

The datasets generated during and/or analysed during the current study are available from the corresponding author on reasonable request.

## Electronic supplementary material

System 1 - no particle displacement

System 3 - particle displacement
